# Buccal Mucosal Graft Urethroplasty

**DOI:** 10.1100/tsw.2010.16

**Published:** 2010-01-08

**Authors:** Angela M. Arlen, Charles R. Powell, Henry T. Hoffman, Karl J. Kreder

**Affiliations:** ^1^ Department of Urology, University of Iowa, Iowa City, IA, USA; ^2^ Department of Otolaryngology, University of Iowa, Iowa City, IA, USA

**Keywords:** urethral stricture, mouth mucosa, transplants, urologic surgical procedures, reconstructive surgical procedures

## Abstract

At our institution, the majority of buccal mucosal graft urethroplasties are performed using a two-team approach with an otolaryngologic surgeon. We report our two-surgeon experience with buccal mucosal grafting for reconstruction of all anterior urethral strictures. Twenty-four men underwent autologous buccal mucosal graft urethroplasty between October 2001 and September 2008 for recurrent urethral stricture disease. Twenty-two underwent a single-stage repair and two underwent a two-stage repair. Medical charts were retrospectively reviewed for demographics, comorbidities, etiology, location and length of stricture, and prior interventions in order to identify predictors of buccal urethroplasty success, defined as no evidence of stricture recurrence. All patients underwent retrograde urethrogram and cystoscopy. Operative and anesthesia times were evaluated. We determined an overall success rate of 83.3% (20 of 24 cases). Mean anesthesia time for single-stage urethroplasty was 155 min and mean operative time was 123 min. One of the two two-stage urethroplasties experienced stricture recurrence (50%). The single-stage buccal graft success rate was 86.4% (19 of 22 cases). Two of the four who developed recurrent stricture disease that required intervention had undergone a previous mesh urethroplasty. Complications developed in four of 24 patients (16.6%), including superficial wound infection (one), superficial wound dehiscence (two), and abscess/fistula formation requiring reoperation (one). The buccal mucosa is an ideal tissue for both single- and two-stage substitution urethroplasty for patients with recurrent stricture disease. Our two-surgeon technique minimizes anesthesia and operative times, and contributes to the overall high success rate and relatively low complication rate.

## INTRODUCTION

Recurrent urethral stricture disease has long posed a significant challenge for urologists. Strictures greater than 2 cm in length often cannot be repaired using end-to-end anastomosis and therefore require substitution urethroplasty. Split- and full-thickness skin grafts, bladder mucosa, and oral mucosa have all been used. Since the initial report of buccal mucosal grafts for the reconstruction of urethral strictures in men in 1992[1], autologous buccal mucosal grafts have been used in repair of strictures greater than 2 cm[2]. Numerous characteristics, including accessibility, resistance to infection, compatibility with a moist environment, and thick epithelium with a thin lamina propria allowing for early inosculation, make the buccal mucosa an ideal tissue for successful urethral reconstruction. The long-term success of buccal mucosal urethroplasty has been documented in several series[3,4,5,6,7,8,9].

At our institution, the vast majority of urethroplasties since 2001 have been performed by a single urologic surgeon (KJK) and buccal mucosal grafts are harvested by a single otolaryngologic surgeon (HTH). This dual-surgeon approach allows for reduced operative time, which is not frequently documented in the urethroplasty literature. We report our ongoing experience with buccal mucosal grafting for the reconstruction of all anterior urethral strictures performed by a single surgeon, including success and complication rates, and operative and anesthesia times.

## MATERIALS AND METHODS

Between October 2001 and September 2008, 24 men underwent buccal mucosal graft urethroplasty for recurrent urethral stricture disease. The average age was 43.2 years (range: 26–71). Twenty-two underwent a single-stage urethroplasty, while the remaining two underwent multistage urethroplasty. Two-stage urethroplasty was reserved for the most difficult cases involving circumferential spongiofibrosis of the urethra, specifically those individuals with complicated cases of hypospadias or lichen sclerosus/balanitis xerotica obliterans (BXO)[10]. Upon Institutional Review Board approval, medical charts were retrospectively reviewed for information regarding patient demographics, comorbidities, etiology, location and length of stricture, and prior interventions in order to identify predictors of buccal urethroplasty success. Anesthesia and operative times were also reviewed. All patients underwent medical history, physical examination, retrograde urethrogram, and cystoscopy prior to urethroplasty.

Successful reconstruction was defined as no recurrent stricture disease requiring intervention, as previously described by Barbagli et al.[8]. Complications, including superficial wound dehiscence, urethrocutaneous fistula, urinary tract infection, wound infection, erectile dysfunction, and donor site complications were also assessed. Average follow-up was 10.5 months.

### Surgical Technique for Single-Stage Urethroplasty

Endotracheal anesthesia was obtained. Patients were placed in the dorsal lithotomy position with all pressure points appropriately cushioned to avoid neurologic sequelae. Specifically, the legs were padded in Allen stirrups. A hand was able to be interposed between the lateral aspect of the tibia and the cushioned boot without undue pressure. If pressure was encountered, the boot was repositioned. Similarly, care was taken to ensure that the sacral spine was supported by the lumbar portion of the bed. Strictures were located with urethroscopy. A midline perineal incision was made, with division of the bulbocavernosus muscle to expose the urethra. Methylene blue was then instilled through the urethra and an 18-French catheter advanced until resistance was met. The urethra was then incised sharply and spatulated at the point of resistance. Proximal sutures were then placed using the J-needle technique.

The buccal mucosal graft was harvested simultaneously. Stensen's duct was carefully identified. An elliptical segment was excised at least 5 mm below the gingivobuccal sulcus and sufficiently below

Stensen's orifice[11]. The graft was then defatted with removal of fibrovascular tissue and fenestrated. Oral hemostasis was achieved using bipolar cautery and closed with interrupted 3.0 vicryl sutures.

The buccal onlay graft was then sutured in place using the previously placed interrupted stitches, as well as a running stitch of 3.0 vicryl on either side of the graft. An 18-French Foley catheter was then advanced into the bladder and the balloon inflated with 10 cc sterile water. Spongioplasty was performed using 3.0 vicryl and the bulbocavernosus muscle was reapproximated using a locking stitch of 3.0 vicryl. A Jackson-Pratt (JP) drain was placed at the closure and brought out through a separate stab incision. The subcutaneous tissues were reapproximated using interrupted stitches of 2.0 vicryl and the skin closed with a running stitch of 4.0 monocryl. All patients were admitted for postoperative observation and average hospital stay was less than 23 h. JP drains were removed prior to discharge in all patients. Follow-up consisted of a pericatheter retrograde urethrogram 3–4 weeks postoperatively to ensure no extravasation prior to catheter removal. All oral care recommendations were discussed with patients prior to discharge, including oral antibiotic coverage for 7 days, germicidal mouthwash four times daily for 2–3 weeks, and soft, mechanical diet for 2–3 weeks. Otolaryngology follow-up was scheduled as needed.

## Surgical Technique for Two-Stage Urethroplasty

As previously stated, the two-stage technique is more reliable than the single stage for circumferential reconstruction of the urethra[10].

Endotracheal intubation and positioning was identical to single-stage urethroplasty. After the stricture was located using urethroscopy and catheter placement, the diseased urethra was sharply incised in the ventral midline. The buccal mucosal grafts were then prepared and quilted into the tunica albuginea. The graft was kept moist, as the perineum was packed with mineral oil-soaked gauze. All patients were admitted following stage 1, with an average hospital stay of less than 23 h, and were instructed to remove dressings in 5 days.

Stage 2 commenced approximately 6 months later, when the buccal mucosa had regained its preoperative characteristics. The graft was tubularized and closed in two layers with dartos fascia comprising the second layer. Skin was then reapproximated. Average operative time for the second stage was 40.5 min, with an average anesthesia time of 59.5 min. Once again, all patients were admitted postoperatively, with an average hospital stay of 23 h. All patients were treated with a 7-day course of oral antibiotics. A pericatheter retrograde urethrogram was performed at the initial postoperative visit at 3–4 weeks.

## RESULTS

Twenty-four patients underwent dorsal onlay buccal graft urethroplasty, with 22 undergoing a single-stage urethroplasty and two undergoing a two-stage procedure. All urethroplasties were performed by a single urologic surgeon (KJK) and the buccal mucosal grafts were harvested by a single otolaryngologic surgeon (HTH). The etiology of urethral stricture disease was identified as infection/BXO in one, hypospadias in one, foreign body in one, suprapubic laser transurethral resection of the prostate in one, trauma in eight (including two with catheter trauma), and unknown in the remaining 12. The mean length of the urethral stricture was 4.1 cm (range: 2–7.5), with a mean length of 4.0 cm in the single-stage urethroplasties and a mean of 5.0 cm in the two-stage procedures. Location of the stricture was within the bulbar urethra in 22 men and the pendulous urethra in two. Mean anesthesia time using this two-surgeon technique was 155 min for single-stage urethroplasty. Mean operative time was 123 min. Mean anesthesia time for the first stage of the two-stage repair was 124.5 min and average operative time was 101 min. The mean anesthesia time for the second stage was 59.5 min and the average operative time was 40.5 min.

Our complication rate was 16.6%, including superficial wound infection treated with oral antibiotics in one, superficial wound dehiscence in two, and abscess/fistula formation requiring reoperation in one. There were no reports of postoperative urinary tract infection or erectile dysfunction. No donor site complications were observed, although all patients experienced temporary trismus postoperatively. There were no reports of lower extremity injury (compartment syndrome or common peroneal nerve paralysis) secondary to prolonged operative positioning. The success rate of all urethroplasties was 83.3% (20 of 24 cases), with success being defined as no additional procedures required. Mean voiding velocity postoperatively was 25.2 cc/sec. One of the men who underwent a two-stage urethroplasty experienced stricture recurrence (50%). The success rate of the single-stage buccal graft was 86.4% (19 of 22 cases). Both men who underwent repair of pendulous urethral stricture had successful repairs; one of these patients had a two-stage repair. The mean time to stricture recurrence was 13.75 months (range: 7–26). Of the four recurrences, three were proximal to the anastomosis of the buccal graft and one was multifocal throughout the graft. Treatment of recurrence included DVIU in three men and daily self-catheterizations in one following Amplatz dilation. None of the four men who developed recurrent stricture disease had any identifiable medical comorbidities that contributed to urethroplasty failure. Both men with a prior history of mesh urethroplasty had stricture recurrence.

## DISCUSSION

The long-term success of autologous buccal graft urethroplasty has been well documented in several series[3,4,5,6,7,8,9]. In 2005, Barbagli et al.[8] reported an 80.4% success rate with single-stage bulbar urethroplasties and in 2007, Levine et al.[3] reported an 81.1% success rate; our data are consistent with these reports. Oral mucosal grafts are known to possess favorable characteristics, with minimal associated morbidity[12,13]. Many of these reports have also commented on the predictors of buccal urethroplasty success; however, there are very little data available on the benefit of the two-surgeon technique and subsequent operative times. Morey and McAninch reported in 1996 that the two-team approach reduced operative time considerably, although their average operative time was not documented[14].

To our knowledge, anesthesia times have not been previously reported upon in the urethroplasty literature. It has been well established that prolonged lithotomy time can lead to serious complications, including lower extremity compartment syndrome and common perineal nerve paralysis[15]. Compartment syndrome, the most severe complication of operative positioning, occurs in the setting of a reperfusion injury to a closed osteofascial compartment. It is well documented that there is a higher risk of compartment syndrome with a prolonged lithotomy position[16,17,18]. Whenever a patient is placed in the lithotomy position for an extended period of time, the surgeon should anticipate the potential for development of a compartment syndrome due to the risks associated with the position[19]. Although these complications are rare, it remains in the patient’s best interest to reduce time spent in the dorsal lithotomy position.

Using the two-surgeon technique, the mean anesthesia time for single-stage urethroplasty in our series was 155 min and mean operative time was 123 min. The mean anesthesia time most accurately reflects the time spent in the dorsal lithotomy position, whereas the reported operative time can be compared to other studies. Mean operative times published for buccal urethroplasty have ranged from 127 to 197 min[4,6,9,20,21].

Our mean follow-up was 10.5 months (range: 3 weeks to 72 months). All patients had an initial postoperative check with pericatheter retrograde urethrogram at approximately 3 weeks. Patients were then scheduled for 3- and 12-month follow-up; however, only 15 patients attended their 3-month appointment with only seven of these men completing their annual visit. The lack of long-term follow-up is a weakness of our study. Cystoscopy was performed postoperatively only if patients experienced symptoms concerning for stricture recurrence.

Two of the four patients who developed recurrent stricture disease had undergone a previous mesh urethroplasty. None of the four men who developed recurrent stricture disease had any significant medical comorbidities that predicted urethroplasty failure. The single patient with a stricture secondary to BXO developed recurrent stricture disease, which is a common finding in the literature[3]. Those strictures that recurred had varying etiologies, including the aforementioned BXO, one with trauma, one following a laser transurethral resection of the prostate, and one of unknown cause. The average stricture length of those cases that failed was 4 cm, slightly lower than the average length of the series (4.1 cm).

## CONCLUSIONS

The buccal mucosa is an ideal tissue for both single- and two-stage substitution urethroplasty. The two-surgeon technique applied at our institution allows for reduced operative time and contributes to the overall low complication rate.
